# Effects of protein supplementation on body composition, physiological adaptations, and performance during endurance training: a systematic review and meta-analysis

**DOI:** 10.3389/fnut.2025.1663860

**Published:** 2025-08-07

**Authors:** Yang Xiao, ZhengWu Deng, Wei Sun, JiaYi Li, WeiFeng Gao

**Affiliations:** ^1^School of Physical Education, Wuhan Sports University, Wuhan, China; ^2^Chinese Volleyball Academy, Beijing Sport University, Beijing, China; ^3^School of Physical Education and Health, East China Normal University, Shanghai, China; ^4^Key Laboratory of Sports Engineering of the General Administration of Sport of China, School of Intelligent Sports Engineering, Wuhan Sports University, Wuhan, China

**Keywords:** endurance training, protein supplements, nutritional supplements, meta-analysis, body composition, aerobic capacity, athletic performance

## Abstract

**Introduction:**

This study aimed to evaluate the long-term effects of endurance training combined with protein supplementation on body composition, physiological adaptations (aerobic/anaerobic capacity), and performance.

**Methods:**

A systematic search was conducted in Web of Science, PubMed, and SPORTDiscus databases on April 16, 2025, using the keywords “endurance training” and “protein supplementation.” Meta-analysis was conducted using a random-effects model, and the main effect size of each outcome was summarized using the standard mean difference (SMD), and moderators were explored using subgroup and regression analyses.

**Results:**

A total of 23 randomized cross-over trials involving 1,146 participants were included. The results showed that protein supplementation during endurance training led to a small, nonsignificant increase in lean body mass (SMD = 0.13, 95% CI: −0.01, 0.28; *p* = 0.07) and a significant improvement in time to exhaustion (TTE) during endurance exercise (SMD = 0.45, 95% CI: 0.15, 0.76; *p* < 0.01). While the overall impact on maximal oxygen uptake (VO2max) was not significant, subgroup analysis revealed that untrained individuals may experience greater improvements in VO2max with protein supplementation (SMD = 0.21). Although aerobic and anaerobic capacities were assessed, protein supplementation did not lead to significant changes in these outcomes, nor did it significantly affect body weight or body fat.

**Discussion:**

In summary, protein supplementation during endurance training appears to modestly enhance endurance performance (TTE) and may offer small benefits for lean body mass, particularly in untrained individuals. However, it does not significantly affect overall body weight, fat mass, or aerobic/anaerobic capacity in the general population.

**Systematic review registration:**

Identifier, CRD420251034453, https://www.crd.york.ac.uk/PROSPERO/recorddashboard.

## Graphical abstract

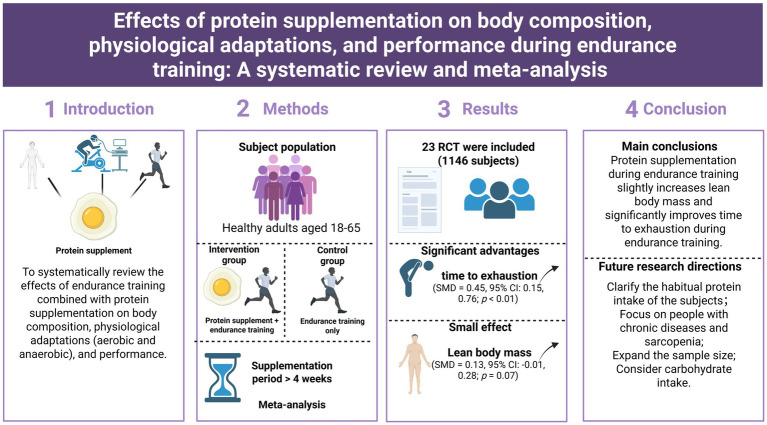

## 1 Introduction

Protein supplementation refers to the increased intake of dietary protein, commonly achieved through commercially available supplements (1). Protein supplementation is popular among fitness enthusiasts, high-performance athletes, and public health advocates for helping individuals meet their protein needs when these requirements are difficult to satisfy through daily food sources alone (2, 3). Although there is now solid evidence that dietary protein intake is fundamental to maximizing muscle hypertrophy and strength during resistance training in strength or power-based athletes (4–6), its role in adaptations to endurance training and optimizing performance in endurance athletes is often overlooked (7).

Positions from the American Dietetic Association, Dietitians of Canada, and the American College of Sports Medicine suggest that optimal nutrition can enhance physical activity, exercise performance, and recovery after exercise (8). Protein supplementation for endurance athletes should prioritize performance adaptations and recovery effects (9, 10). In endurance exercise, carbohydrate and fat oxidation are the main sources of substrate metabolism during endurance exercise (11, 12). Still, as the duration of exercise increases (>2 h), the rate of amino acid oxidation also increases (1), which is equivalent to 6% of the total energy cost of exercise (13), among which branched-chain amino acids (isoleucine, leucine and valine) are oxidized preferentially over other amino acids (14). Protein catabolism in muscle is a key factor in increasing the rate of amino acid oxidation (15), and the intake of branched-chain amino acids alone has been shown to have a positive effect on time trial performance (TT) and peak power (16). Previous meta-analysis evidence shows that the combined intake of protein and carbohydrates improves exhaustion time by an average of 9% (17). This combination stimulates the synthesis rate of skeletal muscle protein in the human body and improves the net protein balance of the whole body (18). The latest evidence shows that protein metabolism during endurance exercise is significantly more active than in the resting state. Exercise intensity is the primary factor affecting protein oxidation, while exercise time and volume have a lesser effect (19). This finding highlights the importance of high protein intake following high-intensity endurance training. According to the current survey, elite endurance athletes appear to implement key pre- and post-training nutritional recovery recommendations (20); however, few athletes deliberately adopt some modern dietary periodization methods (21), suggesting a mismatch between athlete diets and current and developing sports nutrition guidelines. The importance of carbohydrates for endurance training is well established (22, 23), while the role of protein and the adaptive response to endurance training is less clear (7), as the effect of endurance training on skeletal muscle growth is negligible, which may have led to an underestimation of the role of dietary protein in endurance athletes.

The International Society of Sports Nutrition currently recommends that most people who exercise aim for a total daily protein intake in the range of 1.4–2.0 g of protein per kilogram of body weight per day (g/kg/d) (2). The American Dietetic Association, Dietitians of Canada, and the American College of Sports Medicine recommend that endurance athletes consume a total daily protein intake of 1.2–1.7 g/kg/d (8). Endurance athletes habitually consume >1.2 g protein/kg/d according to a 24-h dietary recall, but the distribution throughout the day may not be optimal to maximize skeletal muscle adaptive responses to training (24). To date, Lin et al. are the only researchers who have systematically reviewed the effects of protein supplementation on endurance training adaptations in healthy and clinical populations through meta-analysis (25). They concluded that protein supplementation further increased aerobic capacity, stimulated increases in lean body mass, and improved TT during chronic endurance training in these populations (25). However, their meta-analysis did not explore the potential moderating effects of participant characteristics and protein type on the outcomes (25). Exploring these moderating factors would improve our understanding of how variables such as training status, gender, and protein source (e.g., whey versus other types) influence training adaptations. This knowledge could help develop more tailored nutritional strategies for endurance athletes. In addition, as their search date was up to March 2020, the conclusions of subsequent studies may have challenged their results. For example, Hansen et al. reported that whey protein intake before each exercise session and whey protein and carbohydrate intake after exercise during a six-week endurance training period may enhance the training effect on specific mitochondrial proteins, but did not change aerobic capacity and TT (26). Similarly, Alghannam et al. reported that post-exercise protein supplementation upregulated mTOR expression in skeletal muscle during a six-week endurance training period. However, the magnitude of improvement in maximal oxygen uptake (VO_2_max) was similar between groups (27).

Although protein supplementation for endurance athletes has received extensive attention, there are still some gaps and limitations in the current literature. Compared with individual trials, meta-analyses increase the statistical power of the results (28), provide greater precision and avenues for integrating results, and address methodological differences between studies, such as the age, sex, and training status of participants, the dose of protein consumed, and the duration of the study. Considering that further research is still needed to understand the optimal daily distribution of protein intake, it is unclear whether previous meta-analyses can reflect the conclusions of current studies. Therefore, a comprehensive systematic review and meta-analysis must be conducted to update previous conclusions, derive a higher level of evidence, provide practitioners with the latest integrated results, and provide practical recommendations for endurance athletes and sports nutrition professionals.

The present study aims to systematically review published randomized controlled trials and use meta-analysis to evaluate the effects of endurance training combined with protein supplementation on body composition, physiological adaptations (both aerobic and anaerobic), and performance outcomes, including TT and time to exhaustion (TTE).

In particular, we focus on the interaction between endurance training and protein supplementation to provide robust evidence supporting nutritional strategies for endurance athletes.

## 2 Methods

This systematic review and meta-analysis followed the 2020 PRISMA reporting guidelines (29). To improve research transparency, this study has been pre-registered in the PROSPERO database (registration ID: CRD420251034453).

### 2.1 Information sources and search strategy

Three authors independently conducted a comprehensive literature search across Web of Science Core Collection, PubMed, and SPORTDiscus, covering all available records up to April 16, 2025. The specific date ranges retrieved from each database were: Web of Science (1900–2025), PubMed (1966–2025), and SPORTDiscus (1930–2025). Only peer-reviewed articles were considered, and randomized controlled trials were exclusively included in the search process. For instance, the PubMed search strategy was as follows: (“endurance training” OR “aerobic exercise”) AND (“protein supplementation” OR “whey protein” OR “amino acids”) AND (“exercise performance” OR “VO_2_max” OR “time trial performance”) AND (“body composition” OR “muscle mass” OR “body fat”) AND (“randomized controlled trial” OR “RCT”). The complete search strategies for all databases are provided in Appendix S1. Two snowball search strategies were also applied to enhance comprehensiveness: (1) screening reference lists of eligible studies; and (2) identifying studies that cited the included articles via Google Scholar.

### 2.2 Eligibility criteria

Inclusion criteria were determined according to the Population, Intervention, Control, Outcome, and Study Design (PICOS) framework: (a) Population: healthy adults aged 18 to 65 years, of either sex, with or without prior training experience or protein supplementation; (b) Intervention: nutritional intervention, supplementing the usual diet with protein supplements, regardless of the source of protein; (c) Control: placebo, carbohydrate, or water; (d) Outcome: at least one outcome related to body composition, physiological adaptation, or performance (e.g., body weight, VO_2_max, or TT); (e) Study design: randomized controlled trials, including parallel-group and crossover designs. In addition, we included studies that supplemented with protein during endurance training and studies that combined endurance training with resistance training. Both training modalities aim to improve endurance-related outcomes, and protein may play a role in optimizing adaptations in both contexts. Potential heterogeneity introduced by training modality was addressed through subgroup analyses.

Exclusion criteria were as follows: (1) studies that supplemented with isolated amino acids without whole protein intake; (2) short-term interventions lasting less than 4 weeks; (3) studies that included less than two endurance training sessions per week; (4) nonhuman studies, reviews, research protocols, books, and case reports.

### 2.3 Study selection

The studies identified through database searches were imported into EndNote 21 for management, and duplicates were removed. Two researchers conducted title and abstract screening independently based on the inclusion criteria, excluding irrelevant studies. The full texts of potentially eligible studies were reviewed to confirm inclusion. Disagreements were resolved through discussion or consultation with a third researcher.

### 2.4 Data extraction and transformation

Data extraction and conversion were performed using a standardized process. Two researchers independently extracted information, including authors, publication year, study design, participant characteristics, training regimen, protein supplementation regimen, and outcome measures, and extracted this information into an Excel worksheet. Data extraction mainly included the mean, standard deviation (SD), and sample size before and after the intervention. For studies containing graphical data, the WebPlotDigitizer 4.5^1^ tool was used to extract values. The reliability and validity of this software have been demonstrated (30). If studies only provided data with 95% confidence intervals (CI), they were converted to SD (31):


(1) $$\mathrm{SD}=\sqrt{N}\frac{{\mathrm{CI}}_{\text{high}}-{\mathrm{CI}}_{\mathrm{low}}}{2t}$$

Where *N* is the sample size, CI_high_ is the upper limit of the confidence interval, CI_low_ is the lower limit of the confidence interval, and *t* represents the *t* distribution (Equation 1).

If standard errors were provided, they were converted to SD (Equation 2) (31).


(2) $$\mathrm{SD}=\sqrt{N}\times \mathrm{SE}$$

For studies with missing data, attempts were made to contact the authors to provide information. Data extraction was completed independently by two researchers and cross-validated to ensure accuracy. In case of disagreement, the two researchers resolved it through discussion or negotiation without consulting a third researcher. All data were merged into a unified Excel template.

### 2.5 Study risk of bias assessment

The Cochrane recommended tool ROB2 (32) was used to assess the risk of bias in randomized crossover controlled trials. ROB2 assesses bias in five domains: (D1) randomization, (D2) intervention implementation, (D3) outcome data, (D4) outcome measurement, and (D5) other bias. Each domain was rated as “low,” “moderate,” or “high” risk of bias. The first and second authors performed the assessment, and any disagreements were resolved by discussion with a third reviewer or by consensus.

### 2.6 Statistical analysis

Conventional two-level meta-analysis was performed using the metafor package (33) in R Studio (version 4.4.3). Effect sizes were pooled using the inverse variance weighting method, and heterogeneity was estimated using the restricted maximum likelihood (REML) method. Meta-analysis results were visualized using the orchaRd package (34). Effect sizes were estimated as standardized mean differences (SMD). The standard deviation was the difference between the pre- and post-intervention values (31). The correlation coefficient (“*r*”) was obtained from the article first, but most articles did not provide the correlation coefficient value, so a conservative value of 0.5 was taken as the correlation coefficient value, as recommended by the Cochrane Handbook (31). To test the robustness of the results, sensitivity analyses were also conducted using *r* = 0.3 and *r* = 0.7 (Equation 3).


(3) $$\begin{array}{c} {\mathrm{SD}}_{\text{change}}=\sqrt{{\mathrm{SD}}_{\mathrm{pre}}^{2}+{\mathrm{SD}}_{\text{post}}^{2}-(2\times r\times {\mathrm{SD}}_{\mathrm{pre}}\times {\mathrm{SD}}_{\text{post}})} \end{array}$$

Effect sizes were classified as slight (0.2), small (0.2–0.5), moderate (0.5–0.8), or large (>0.8) (35). Statistical significance was set at *p* < 0.05. Cochran’s Q test and *I^2^* statistic were used to assess heterogeneity, but *I^2^* is the most commonly used and widely recommended indicator (36), so we will mainly report *I^2^*, with *I^2^* values of 25, 50, and 75% indicating low, moderate, and high heterogeneity, respectively (37). *I^2^* > 50% indicated significant heterogeneity. Sensitivity analysis was performed using the leave-one-out method to assess the impact of each study on the effect size.

To further explore the sources of heterogeneity and potential moderators, subgroup and regression analyses were performed. Subgroups included training status (trained or untrained) (38), intervention duration (≤8 weeks or >8 weeks), sex (male only or female only or mixed), protein type (whey protein or other protein), and training method (endurance training or concurrent training). In addition to subgroup analyses of dichotomous variables, we also performed regression analyses on continuous variables such as daily protein intake, intervention duration, total intervention duration, and age. Publication bias was assessed for studies with at least 10 data points using funnel plots (39) and Egger’s test (40), with *p* > 0.05 indicating no bias. For outcomes with fewer than 10 data points (41), a leave-one-out analysis was performed to evaluate the impact of individual studies on the overall pooled effect.

### 2.7 Certainty of evidence assessment

We evaluated evidence quality using the GRADE approach, examining five domains (42): risk of bias, inconsistency, indirectness, imprecision, and publication bias. Two authors independently rated evidence as high, moderate, low, or very low. Disagreements were resolved through discussion with a third author.

## 3 Results

### 3.1 Study selection

**Figure 1:**
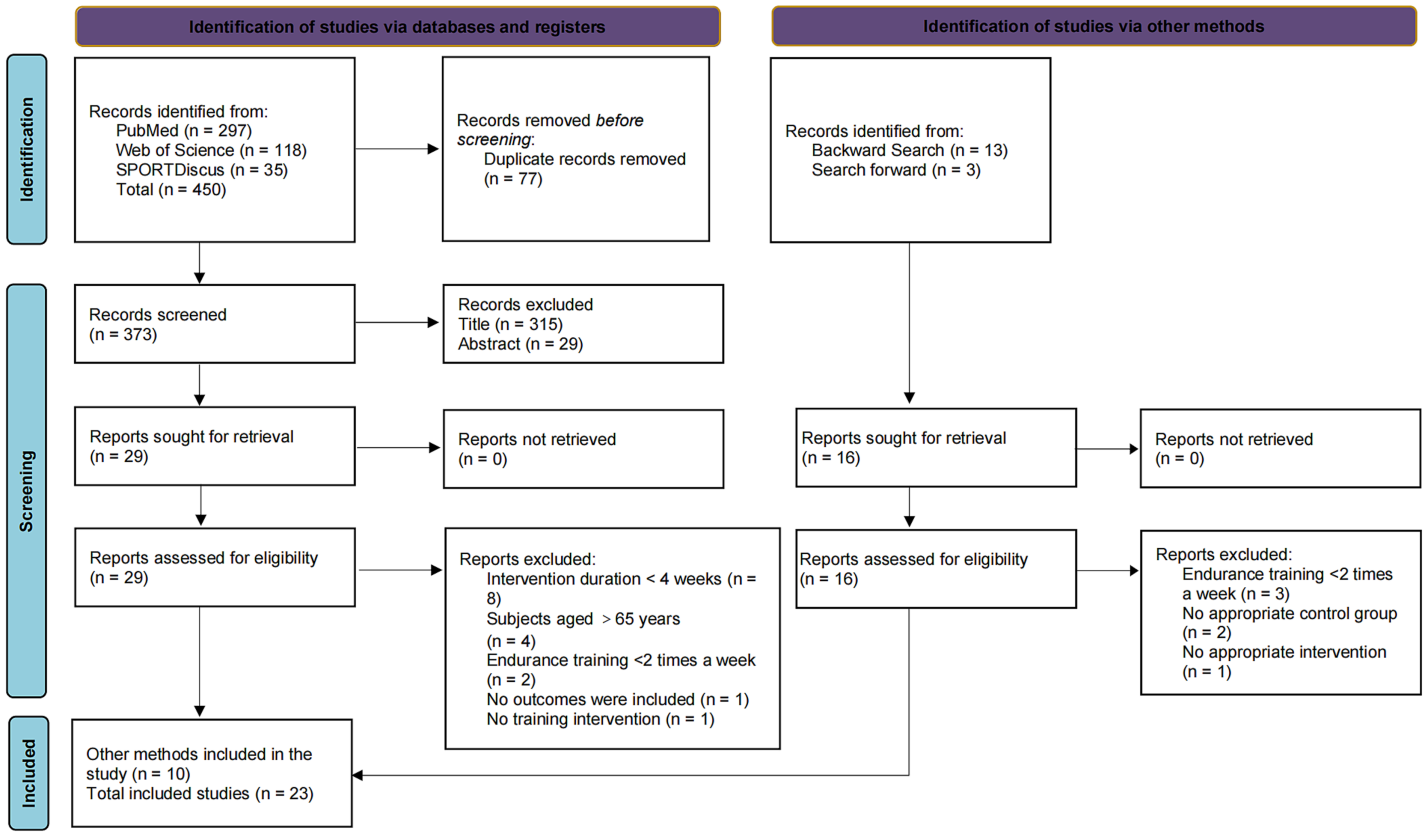
Literature screening flow chart.

We conducted a preliminary search of PubMed, Web of Science, and SPORTDiscus, which retrieved a total of 450 articles. After repeated literature screening, 373 articles remained. After screening the titles and abstracts, 29 articles were evaluated in full text. Some articles were excluded due to insufficient intervention period, inconsistent age of subjects, etc., leaving 13 articles in the preliminary search. In addition, 16 relevant articles were found through forward and backward searches of Google Scholar. After full-text review, 6 studies were excluded due to inconsistent study designs: 2 studies were excluded because the frequency of the intervention period was less than twice a week; 1 study lacked a suitable control group; and 1 study did not implement a suitable endurance training program. A total of 23 articles were included. The screening process is shown in Figure 1.

### 3.2 Characteristics of the studies included

Twenty-three studies were included in this review, all of which were randomised controlled trials, one of which was a crossover trial (43). Fifteen studies were double-blind (65%) and eight studies were single-blind or did not report blinding (35%). The studies involved 1,146 participants, including 897 men (78%) and 249 women (22%). The sample size of the individual studies ranged from 6 to 387 participants, with an age range of 18 to 63.5 years, and only two studies had participants aged 60 to 65 years (44, 45). Participants were divided into trained (757 participants) and untrained (389 participants) groups according to their physical activity level, with two studies involving military personnel and one study involving sports students, who were considered trained. In terms of training programs, 12 studies were concurrent training (43, 45–55), 11 studies were single endurance training (26, 27, 44, 56–63), and 7 studies had a gradual increase in exercise intensity (27, 48, 52, 54–56, 59). The duration of training intervention ranged from the shortest 6 weeks to the longest 26 weeks, and the intervention duration of most studies was between 8 and 12 weeks. In terms of protein supplement types, 11 studies used whey protein, 4 studies used soy protein, 2 studies used milk protein, 2 studies used casein, 2 studies used collagen peptides, 1 study used amino acid supplements, and 1 study used beef protein. The daily protein intake in a single study ranged from 0.96 g/kg/day to 3.8 g/kg/day (52, 53), and the daily protein intake in most studies ranged from 1.2 g/kg/day to 2.3 g/kg/day. Six studies did not report daily protein intake. For body composition measurement, 9 studies used dual-energy X-ray absorptiometry (DXA), 5 studies used bioelectrical impedance analyzer (BIA), 2 studies used skinfold thickness measurement, 1 study used air displacement plethysmography (ADP), and 1 study used underwater weighing (UWW). Other details are shown in Table 1.

**Table 1:** Essential characteristics of the included literature.

Study	Study design	Participant	Training type	Training program	Protein supplement group	Placebo/Control group	Related results
Antonio et al. (46)	Parallel RCT Double-blind	N = 19, Men Ration = 0% Age = 26.9 ± 6.1/27.4 ± 7.4 years BW = 68.9 ± 15.2/74.0 ± 12.8 kg Healthy untrained women	CT	Training time: 6 weeks ET: 3 times a week, 20 min each time Intensity: 70%HRmax RT: 3 times a week	Amino acid supplement (1.24 g protein/kg/day) Intake time: 20 min before and 20 min after training Average daily protein intake per kg: 1.24	Taking the same number of identical-looking pills containing fiber (0.9 g protein/kg/day). Average daily protein intake per kg: 0.9	Body composition (DXA), TTE
Flakoll et al. (47)	Parallel RCT Double-blind	N = 387, Men Ration = 100% Age = 18.9 ± 0.1 years BW = 74.4 ± 14.5/74.9 ± 13.7 kg Marine Corps Recruits	CT	Training time:54 days ET: 24 days of running and 3 days of walking training Intensity: n/a RT: Push-ups, sit-ups, pull-ups	Whey protein: 10 g protein, 8 g carbs, and 3 g fat Intake time: post-exercise intake Average daily protein intake per kg: n/a	CON1: nonnutritive placebo tablets CON2: contains 8 grams of carbohydrates and 3 grams of fat Average daily protein intake per kg: n/a	Body composition (BIA)
Lockwood et al. (48)	Parallel RCT Blinding not reported	N = 28, Men Ration = 50% Age = 34.8 ± 4.9/32.6 ± 6.0 years BW = 82.3 ± 15.3/84.7 ± 20.6 kg VO_2_max = 32.9 ± 7.1/35.7 ± 10.1 mL/kg/min Sedentary obese people	CT ( gradually increase the amount and intensity of exercise)	Training time:10 weeks ET: 15 to 35 min per day, 3 days per week Intensity: 40 to 70% HRmax RT: 2 days a week	Whey protein (1,854 kJ/day, 131 g protein/day, 190 g carbohydrates/day, 63 g fat/day, 22.9 g dietary fiber/day) Intake time: subject decision Average daily protein intake per kg: 1.58	No additional dietary protein supplementation (1,951 kJ/day, 74.9 g protein/day, 253 g carbohydrate/day, 70.7 g fat/day, 15.5 g dietary fiber/day) Average daily protein intake per kg: n/a	Body composition (DXA), performance, aerobic capacity
Walker et al. (49)	Parallel RCT Double-blind	N = 30, Men Ration = 100% Age = 26.9 years BW = 86.8 ± 16.4/83.0 ± 7.7 kg Air Force, exercise at least 3 times a week	CT	Training time:8 weeks ET: run at least 3 times a week. Intensity: n/a RT: At least 2 h per week	Whey protein (112 kcal/serving, 19.7 g protein/serving) Intake time: before and after exercise Average daily protein intake per kg: 1.07	Placebo, 0 g protein Average daily protein intake per kg: 1.07	Body composition (DXA), performance
Ferguson-Stegall et al. (56)	Parallel RCT Double-blind	N = 32, Men Ration = 50% Age = 22.0 ± 0.5 years BW = 71.7 ± 13.6 kg Recreationally active, but untrained men and women	ET (gradually increase the intensity of exercise)	Training time:4 weeks ET: Cycling for 30 to 60 min, 5 times per week Intensity: 75 to 80% of VO_2_max	Milk protein (11.48 g carbohydrates/100 mL, 3.67 g protein/100 mL, 2.05 g fat/100 mL, 79.05 kcal/100 mL) Intake time: immediately and 1 h after exercise Average daily protein intake per kg: n/a	CON1: carbohydrate drink (15 g carbs/100 mL, 0 g protein, 2.05 g fat/100 mL, 79.05 kcal/100 mL) CON2: non-caloric placebo Average daily protein intake per kg: n/a	Body composition (DXA), aerobic capacity
Berg et al. (57)	Parallel RCT Blinding not reported	N = 30, Men Ration = 67% Age = 24.0 ± 2.1/23.3 ± 1.6 years BW = 67.5 ± 10.6/68.3 ± 12.3 kg Sports students	ET	Training time:6 weeks ET: 60 min of endurance training per day, 5 times per week Intensity: aerobic threshold	Soy protein: contains 53.3 grams of protein per serving, take 2 servings daily Intake time: immediately and 1 h after exercise Average daily protein intake per kg: n/a	No supplement Average daily protein intake per kg: n/a	Body composition (ADP), performance
Cramer et al. (58)	Parallel RCT Double-blind	N = 32, Men Ration = 100% Age = 23 ± 3 years mL/kg/min BW = 90.7 ± 11.2/85.1 ± 12.8 kg Healthy and physically active men	ET	Training time:8 weeks ET: 60 min of cycling 5 times a week Intensity: 70%VO_2_max	Whey protein drinks: contain 14 grams of protein Intake time: immediately after training Average daily protein intake per kg: n/a	Placebo Average daily protein intake per kg: n/a	Body composition (UWW), performance, aerobic capacity
Gryson et al. (45)	Parallel RCT Double-blind	N = 48, Men Ration = 100% Age = 60.8 ± 2.7 years BW = 81.1 ± 10.1 kg Elderly sedentary men	CT	Training time:16 weeks ET: 3 × 6 min of aerobic exercise, 3 times a week Intensity: 50 to 80%VO_2_max RT: 3 × 6 min of free weights, 3 times per week	Milk protein: contains 10 g of milk protein Intake time: at breakfast and after exercise Average daily protein intake per kg: n/a	Placebo drink containing 4 g of dairy protein Average daily protein intake per kg: n/a	Body composition (DXA), aerobic capacity
McAdam et al. (50)	Parallel RCT Double-blind	N = 69, Men Ration = 100% Age = 19 ± 1 years BW = 73.4 ± 12.7/72.3 ± 10.9 kg Male Army Soldier	CT	Training time:9 weeks Troop physical training	Whey protein: contains 38.6 grams of protein per serving, take two servings daily (2.8 ± 0.5 g/kg/day) Intake time: after training and before bed Average daily protein intake per kg: 2.8	Energy-matched carbohydrates (129 g/day) Average daily protein intake per kg: n/a Average daily protein intake per kg: 1.6	Body composition (skinfold), performance
Ormsbee et al. (51)	Parallel RCT Blinding not reported	N = 51, Men Ration = 51% Age = 21 ± 0.6/20.3 ± 0.5 years BW = 81.7 ± 1.6/73.1 ± 1.4 kg Sedentary healthy people	CT	Training time:26 weeks ET: run 2 to 3 times a week for 25 to 40 min each time Intensity: 70%HRmax RT: 2 to 3 resistance training sessions per week	Whey protein: each serving contains 280 kcal, 42 g protein, 21 g carbohydrates, and 1.5 g fat, take twice daily (2.3 g/kg/day) Intake time: immediately after exercise and 8 to 12 h later Average daily protein intake per kg: 2.3	Contains calorie-equivalent carbohydrates (70 g sucrose and fructose) and an equivalent blend of vitamins and minerals Average daily protein intake per kg: 1.0	Body composition (DXA), aerobic capacity
Knuiman et al. (59)	Parallel RCT Single-blind	N = 40, Men Ration = 100% Age = 22.5 ± 2.3/21.5 ± 1.7 years BW = 77.2 ± 7.3/72.3 ± 5.7 kg Recreationally active young men	ET (gradually increase the intensity of exercise)	Training time:12 weeks ET: 60 min of cycling 3 times a week Intensity: n/a	Casein (127 kcal/250 mL, 28.7 g protein/250 mL, 0.3 g fat/250 mL, 2.7 g carbohydrate/250 mL) Intake time: after exercise and before bed Average daily protein intake per kg: 1.8	Carbohydrates (129 kcal/250 mL, 0.6 g protein/250 mL, 2.4 g fat/250 mL, 26.3 g carbohydrates/250 mL) Average daily protein intake per kg: 1.3	Body composition (DXA), aerobic capacity, performance
Naclerio et al. (60)	Parallel RCT Double-blind	N = 25, Men Ration = 100% Age = 30.3 ± 8.8/34.1 ± 7.8 years BW = 68.9 ± 4.4/66.2 ± 4.0 kg Endurance athletes	ET	Training time:10 weeks ET: train 5–6 times a week, including HIIT Intensity: polarized intensity	24 g beef and whey orange drink (204 kcal/250 mL, 27.7 g carbs, 19.84 g protein) Intake time: post-training supplement Average daily protein intake per kg: 2.1	Carbohydrate drink (204 kcal/250 mL, 50.1 g carbs, 0.40 g protein) Average daily protein intake per kg: 1.9	Body composition (DXA), aerobic capacity, performance
Jonvik et al. (61)	Parallel RCT Double-blind	N = 56, Men Ration = 100% Age = 28 ± 6/26 ± 6 years BW = 82.0 ± 9.6/79.7 ± 13.9 kg Healthy males	ET	Training time:12 weeks ET: 1 60-min cycling session, 1 SIT (6 × 1-min), 1 HIIT (6 × 4-min) per week Intensity: 75 to 95% HRmax	Casein (129 kcal/250 mL, 28.7 g protein, 0.3 g fat, 25.8 g carbohydrates) Intake time: after exercise and before bed Average daily protein intake per kg: 1.58	Placebo (129 kcal/250 mL, 0.6 g protein, 2.4 g fat, 25.8 g carbohydrate) Average daily protein intake per kg: 1.18	Body composition (DXA), aerobic capacity, performance
Jendricke et al. (52)	Parallel RCT Double-blind	N = 60, Men Ration = 0% Age = 25.4 ± 4.2/26.8 ± 5.7 years BW = 62.5 ± 8.6/63.3 ± 6.0 kg Recreational sportive female runner	CT (gradually increase the intensity of exercise)	Training time:12 weeks ET: run for 1 h 3 times a week Intensity: 80 to 90% VIAT RT: 3 times a week	15 g collagen peptides Intake time: before and after training Average daily protein intake per kg: 0.96	15 g silicon dioxide Average daily protein intake per kg: 0.95	Body composition (BIA), performance
Forbes et al. (53)	Parallel RCT Blinding not reported	N = 31, Men Ration = 52% Age = 27 ± 4/26 ± 3 years BW = 65.9 ± 13.3/82.5 ± 9.1 kg Men and women trained in rowing	CT	Training time:6 weeks ET: 4 rowing sessions per week, including HIIT Intensity: ventilatory threshold RT: 2 times a week	Intervention group 1: whey protein isolate Intervention group 2: whey protein concentrate Intake time: every morning Average daily protein intake per kg: 3.8 grams for men, 3.2 grams for women	Placebo Average daily protein intake per kg: n/a	Performance, aerobic capacity
Hansen et al. (26)	Parallel RCT Double-blind	N = 24, Men Ration = 92% Age = 30 ± 9/31 ± 10 years BW = 70.1 ± 7.7/74.1 ± 7.4 kg Trained runners	ET	Training time: 6 weeks ET: 5 to 7 endurance training sessions per week, including HIIT Intensity: pyramid distribution	Whey protein (0.3 g protein/kg, 1 g carbohydrate/kg) Intake time: before and after exercise Average daily protein intake per kg: 1.59	Carbohydrates (1.3 g carbs/kg) Average daily protein intake per kg: n/a	Body composition (BIA), aerobic capacity, performance
Alghannam et al. (27)	Parallel RCT Double-blind	N = 25, Men Ration = 92% Age = 20 ± 2 years BW = 76.3 ± 12 kg Healthy men and women	ET (gradually increase the intensity of exercise)	Training time: 6 weeks ET: 40 to 60 min of running Intensity: 70 to 75% VO_2_max	Whey protein (0.8 g protein/kg, 1.6 g carbohydrate/kg) Intake time: immediately and 1 h after exercise Average daily protein intake per kg: 2.3	Carbohydrates (1.6 g carbs/kg) Average daily protein intake per kg: 1.4	Aerobic capacity
Hsu et al. (44)	Parallel RCT Blinding not reported	N = 46, Men Ration = 9% Age = 57.1 ± 3.8/58.6 ± 3.8 years BW = 71.8 ± 11.9/67.1 ± 11.0 kg Male elite triathlete	ET	Training time: 12 weeks ET: exercise 3 times a week for 60 min, including HIIT Intensity: 70 to 90% HRmax	Soy protein (0.4 g/kg/day) Intake time: immediately and 1 h after exercise Average daily protein intake per kg: 1.6	No supplements Average daily protein intake per kg: 0.91	Body composition, aerobic capacity, and performance
Valenzuela et al. (43)	Crossover RCT Double-blind	N = 6, Men Ration = 100% Age = 21 ± 3 years BW = 66 ± 4 kg Obese middle-aged people	CT	Training time: 8 weeks ET: triathlon training program Intensity: 70 to 90% HRmax RT: 1–2 resistance training sessions per week	25 g beef supplement (99.33 kcal, <0.5 g carbs, 20.5 g protein) Intake time: immediately after training Average daily protein intake per kg: 2.25	27.1 g carbohydrate supplement (99.9 kcal, 19.3 g carbs, 2.0 g protein) Average daily protein intake per kg: 1.89	Body composition (skinfold)
Röhling et al. (62)	Parallel RCT Blinding not reported	N = 23, Men Ration = 70% Age = 29.0 ± 11.0/28.6 ± 8.7 years BW = 73.7 ± 6.7/71.1 ± 7.5 kg Experienced endurance runners	ET	Training time: 12 weeks ET: specific plan unknown Intensity: unknown	Soy protein supplement (217 kcal, 27.2 g protein, 24.6 g carbohydrate, 1.0 g fat) Intake time: once in the morning and once in the evening Average daily protein intake per kg: 2.25	Not supplemented Average daily protein intake per kg: n/a	Body composition (skinfold), aerobic capacity, and performance
Li et al. (63)	Parallel RCT Blinding not reported	N = 16, Men Ration = 0% Age = 38.00 ± 5.88/34.25 ± 5.34 years BW = 55.69 ± 7.30/59.06 ± 11.72 kg Healthy sedentary women	ET	Training time: 8 weeks ET: 60 min of aerobic endurance training twice a week Intensity: 40 to 65% HRmax	20 g isolate soy protein supplement (72 kcal, 11.6 g protein, 0.4 g fat, 5.5 g carbohydrates) Intake time: within 30 min after training Average daily protein intake per kg: n/a	Hydration only Average daily protein intake per kg: n/a	Body composition (skinfold)
Jerger et al. (54)	Parallel RCT Double-blind	N = 32, Men Ration = 100% Age = 28.6 ± 5.0/28.3 ± 5.6 years BW = 78.5 ± 9.2/75.6 ± 7.7 kg Endurance-trained men	CT (gradually increase the intensity of exercise)	Training time: 12 weeks ET: 60-min running sessions 3 times a week Intensity: 80 to 90% VIAT RT: 3 times a week resistance training	15 g collagen peptides Intake time: before and immediately after training Average daily protein intake per kg: 1.04	15 g silica placebo Average daily protein intake per kg: 1.29	Body composition (BIA), performance
Reljic et al. (55)	Parallel RCT Double-blind	N = 36, Men Ration = 42% Age = 26 ± 4/27 ± 6 years BW = 65.9 ± 11.16/75.9 ± 13.7 kg Untrained healthy people	CT (gradually increase the intensity of exercise)	Training time: 8 weeks ET: 14-min HIIT (5 × 1-min) 2 times a week Intensity: 80 to 95% HRmax RT: total body resistance training 5 times a week	40 g whey protein supplement (213 kcal, 40 g protein, 7.5 g carbohydrate, 2.6 g fat) Intake time: after exercise Average daily protein intake per kg: 1.2	Maltodextrin placebo (222 kcal, 5 g protein, 46 g carbohydrate, 2 g fat) Average daily protein intake per kg: 1.1	Body composition (BIA), aerobic capacity

BW, body weight; CT, Concurrent training; ET, Endurance training; RT, Resistance training; HIIT, high-intensity interval training; BIA, Bioelectrical Impedance Analyzer; DXA, dual-energy X-ray absorptiometry; EAA, essential amino acids; TTE, Time to Exhaustion; ADP, Air Displacement Plethysmography; UWW, underwater weighing; VIAT, Individual anaerobic threshold rate; HRmax, Maximum heart rate.

### 3.3 Effect size of the intervention

#### 3.3.1 Body composition

**Figure 2:**
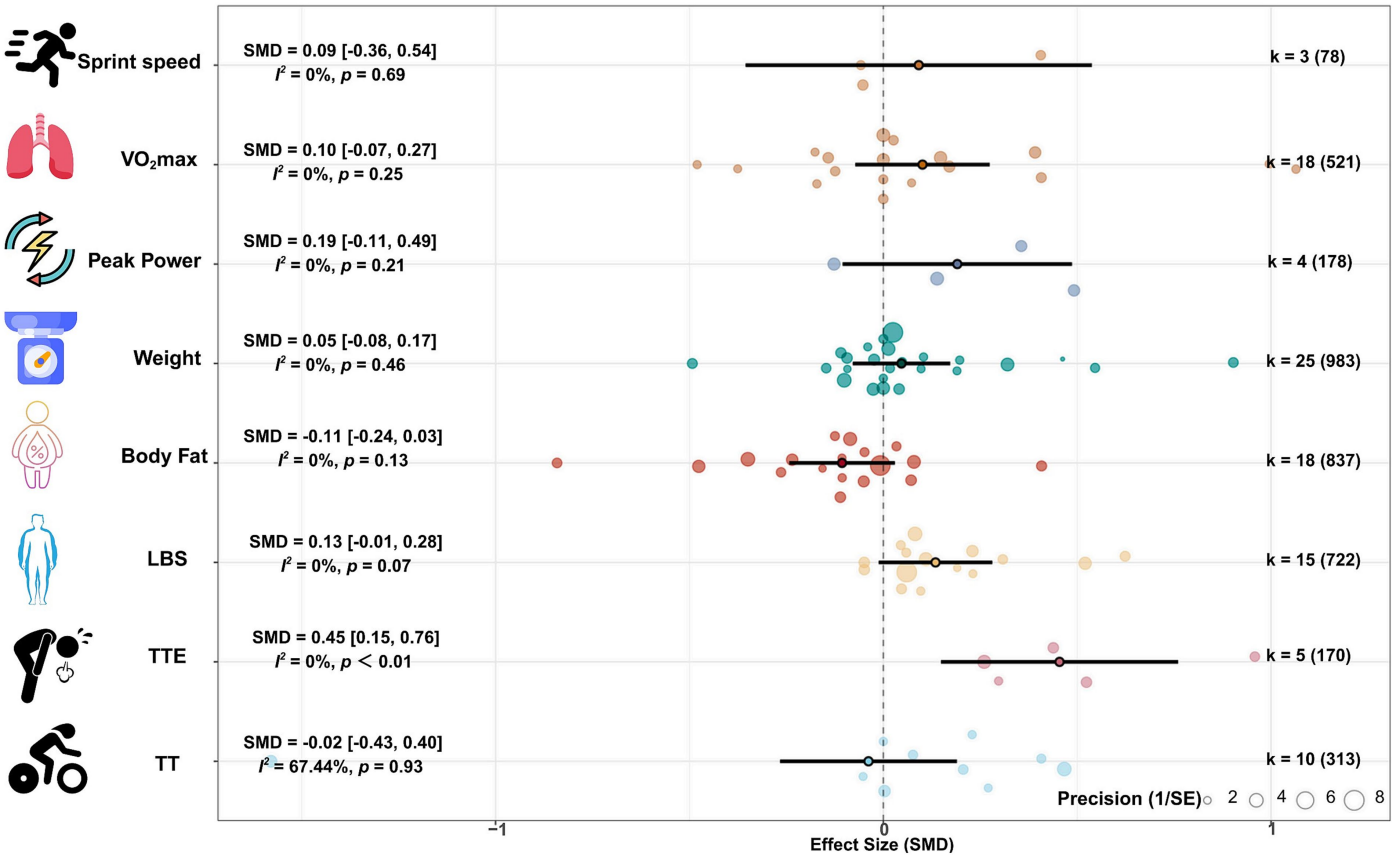
Forest plot of combined effect size.

The meta-analysis results on body weight (25 studies, 983 participants) and body fat (18 studies, 837 participants) showed that protein supplementation had no significant effect on body weight (SMD = 0.05, 95% CI: −0.08, 0.17; *p* = 0.46; *I*^2^ = 0%) and body fat (SMD = −0.11, 95% CI: −0.24, 0.03; *p* = 0.13; *I*^2^ = 0%), and the pooled effect heterogeneity was low. The meta-analysis results on lean body mass showed that protein supplementation may have a small effect on increasing lean body mass (SMD = 0.13, 95% CI: −0.01, 0.28; *p* = 0.07; *I*^2^ = 0%), and the pooled effect heterogeneity was low. See Figure 2.

#### 3.3.2 Physiological adaptation

Meta-analysis results of aerobic capacity (18 studies, 521 participants) and anaerobic capacity (4 studies, 178 participants) showed that protein supplementation had no significant effect on VO_2_max (SMD = 0.10, 95% CI: −0.07, 0.27; *p* = 0.25; *I*^2^ = 0%) and peak power (SMD = 0.05, 95% CI: −0.11, 0.49; *p* = 0.21; *I*^2^ = 0%), with low heterogeneity of the combined effect size. See Figure 2.

#### 3.3.3 Performance

The meta-analysis results of time to exhaustion (TTE) (5 studies, 170 participants) showed that protein supplementation could significantly prolong TTE (SMD = 0.45, 95% CI: 0.15, 0.76; *p* < 0.01; *I*^2^ = 0%), with a moderate effect size and low heterogeneity. The meta-analysis results of TT (10 studies, 313 participants) and sprint speed (3 studies, 78 participants) showed that protein supplementation had no significant effect on TT (SMD = −0.02, 95% CI: −0.43, 0.40; *p* = 0.93; *I*^2^ = 67.44%) and sprint speed (SMD = 0.09, 95% CI: −0.36, 0.76; *p* = 0.69; *I*^2^ = 0%), and the heterogeneity of the combined effect of TT was large. See Figure 2.

### 3.4 Moderator analysis

**Figure 3:**
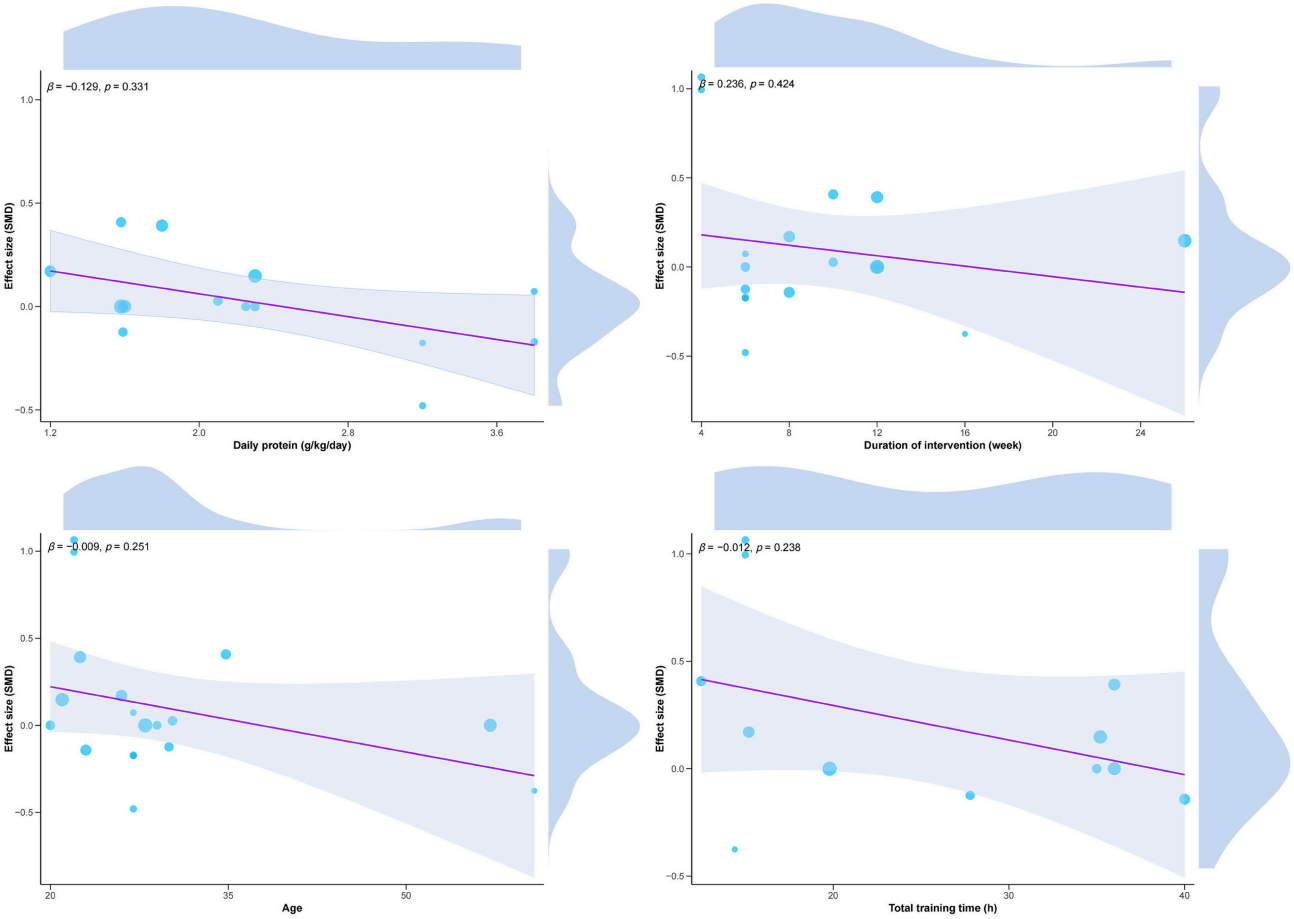
Regression analysis of VO_2_max.

We conducted subgroup analyses of VO_2_max and lean body mass to explore potential moderating factors of the intervention effect. See Tables 2, 3. The results showed that no significant moderating effect was observed in all subgroups (*p* > 0.05), but in terms of VO_2_max, the subjects who did not perform protein supplementation training (SMD = 0.21) had a better improvement in VO_2_max than those who performed protein supplementation training. There was a statistically marginal effect (*p* = 0.06). In addition, we performed regression analysis to examine the moderating effect of age, daily protein intake, total intervention duration, and number of intervention weeks on the combined effect of VO_2_max; however, no significant moderating effect was observed (*p* > 0.05). See Figure 3.

**Table 2:** Subgroup analyses based on meta-analyses results of VO_2_max.

Subgroup	K (*N*)	SMD	95%CI	*p*_d_	*I* ^2^	*p*_b_
Training status						
Untrained	10 (329)	0.21	[−0.01, 0.43]	0.06	0%	0.10
Trained	8 (192)	−0.09	[−0.37, 0.20]	0.56	0%	
Duration of intervention						
≤8 weeks	10 (234)	0.11	[−0.17, 0.38]	0.45	0%	0.97
>8 weeks	8 (287)	0.10	[−0.13, 0.33]	0.41	0%	
Sex						
Mixed sex	9 (276)	0.23	[−0.01, 0.47]	0.06	0%	0.21
Male only	7 (208)	0.01	[−0.26, 0.29]	0.92	0%	
Female only	2 (37)	−0.33	[−0.98, 0.32]	0.32	0%	
Protein type						
Whey protein	14 (356)	0.10	[−0.11, 0.31]	0.33	0%	0.96
Other proteins	4 (165)	0.09	[−0.21, 0.40]	0.55	0%	
Training method						
ET	10 (314)	0.15	[−0.07, 0.38]	0.17	0%	0.45
CT	8 (207)	0.02	[−0.25, 0.29]	0.89	0%	

SMD: pooled effect size between the effects observed in the protein supplementation group and the control group; *p*_d_: overall pooled effect; *p*_b_: between-subgroup difference. ET, endurance training; CT, concurrent training.

**Table 3:** Subgroup analyses based on meta-analyses results of LBM.

Subgroup	K (*N*)	SMD	95%CI	*p*_d_	*I* ^2^	*p*_b_
Training status						
Untrained	9 (248)	0.19	[−0.07, 0.44]	0.15	0%	0.62
Trained	6 (474)	0.10	[−0.07, 0.29]	0.24	0%	
Duration of intervention						
≤8 weeks	7 (400)	0.10	[−0.10, 0.29]	0.34	0%	0.57
>8 weeks	8 (322)	0.18	[−0.04, 0.40]	0.10	0%	
Sex						
Mixed sex	5 (142)	0.24	[−0.12, 0.60]	0.19	0%	0.82
Male only	8 (505)	0.11	[−0.06, 0.29]	0.21	0%	
Female only	2 (75)	0.13	[−0.33, 0.58]	0.58	0%	
Protein type						
Whey protein	8 (513)	0.14	[−0.03, 0.32]	0.11	0%	0.85
Other proteins	7 (209)	0.11	[−0.16, 0.38]	0.42	0%	
Training method						
ET	4 (113)	0.16	[−0.21, 0.53]	0.39	0%	0.87
CT	11 (609)	0.13	[−0.03, 0.29]	0.11	0%	

SMD: pooled effect size between the effects observed in the protein supplementation group and the control group; *p*_d_: overall pooled effect; *p*_b_: between-subgroup difference. ET, endurance training; CT, concurrent training; LBM, Lean body mass.

### 3.5 Risk of bias

#### 3.5.1 Bias and methodological issues

Among the studies with random allocation, except for the study by Jonvik et al. (61), which had a low overall risk of bias, the remaining studies had some concerns. Most studies (74%) did not disclose the details of the randomization process and allocation, resulting in a moderate risk of bias in the randomization process. In addition, there was insufficient compliance control during the intervention period, or the subjects were not blinded (65%), resulting in a risk of bias in the intervention process. The risk of bias for each study is shown in Appendix S2.

Based on the PEDro scale, the quality of the included studies was generally good. Of the 23 studies, 18 scored ≥6, which was of medium or higher quality, and only 5 scored 5. Most studies performed well in terms of random allocation, baseline comparability, and key outcome reporting, but there were still deficiencies in blind control. Specific scores are shown in Appendix S3.

#### 3.5.2 Publication bias

We drew funnel plots for outcome indicators with 10 or more studies (see Appendix S4). Egger’s test results showed that there was no significant publication bias for all indicators (*p* > 0.05).

### 3.6 Sensitivity analysis

We used the leave-one-out method to conduct a sensitivity analysis of the effect size of each study. The results showed that after excluding the study of Jovik et al. (61). In the TT indicator, the heterogeneity was significantly reduced (SMD = 0.22, *I*^2^ = 0%, *p* = 0.078), but further exploration found that the design of this study was not significantly different from that of other studies, so it was retained. The combined effect size of the other outcome indicators showed stability in the sensitivity analysis.

In addition, sensitivity analyses were performed using alternative assumed correlation coefficients (*r* = 0.3 and *r* = 0.7), which did not lead to substantial changes in the results, indicating that the findings were robust to the choice of correlation coefficient.

### 3.7 Quality of evidence

Based on the GRADE assessment, all indicators were downgraded due to a certain risk of bias in the included studies. Except for TTE, which had a moderate quality of evidence, the quality of evidence for the other indicators was low or very low. See Table 4.

**Table 4:** Level of evidence for article conclusions based on GRADE.

Outcome	No of participants (studies)	Certainty assessment					Standardized mean effect (95%CI)	Grade
		Risk of bias	Inconsistency	Indirectness	Imprecision	Other		
Protein supplements vs. placebo								
Weight	983 (25 RCT)	Serious	Not serious	Not serious	Serious	None	0.05[−0.08, 0.17]	⨁⨁◯◯ Low
Body fat	837 (18 RCT)	Serious	Not serious	Not serious	Serious	None	−0.11[−0.24, 0.03]	⨁⨁◯◯ Low
Lean body mass	722 (15 RCT)	Serious	Not serious	Not serious	Serious	None	0.13[−0.01, 0.28]	⨁⨁◯◯ Low
VO_2_max	521 (18 RCT)	Serious	Not serious	Not serious	Serious	None	0.10[−0.07, 0.27]	⨁⨁◯◯ Low
TT	313 (10 RCT)	Serious	Serious	Not serious	Serious	None	−0.02[−0.43, 0.40]	⨁◯◯◯ Very low
TTE	170 (5 RCT)	Serious	Not serious	Not serious	Not serious	None	0.45[0.15, 0.76]	⨁⨁⨁◯ Moderate
Peak Power	178 (4 RCT)	Serious	Not serious	Not serious	Serious	None	0.19[−0.11, 0.49]	⨁⨁◯◯ Low
Sprint speed	78 (3 RCT)	Serious	Not serious	Not serious	Serious	None	0.09[−0.36, 0.54]	⨁⨁◯◯ Low

GRADE quality of clinical evidence and recommendations: high: the research group is very confident in the estimated effect size; medium: the research group has moderate confidence in the estimated effect size; low: the research group has limited confidence in the estimated effect size; very low: the research group has very limited confidence in the estimated effect size.

## 4 Discussion

This article included 23 studies (1,146 subjects) for meta-analysis. The comprehensive results showed that, in terms of body composition, long-term protein supplementation appeared to have a marginal benefit in increasing lean body mass, with a low level of evidence. In terms of performance, long-term protein supplementation helped prolong TTE in endurance exercise, with a moderate level of evidence. Although the overall subgroup analysis and meta-regression analysis did not show a significant moderating effect, the training status of the subjects showed a potential moderating effect in VO_2_max.

### 4.1 Body composition to protein supplementation

Our meta-analysis results showed that protein supplementation had no significant effect on body weight and body fat during endurance training, while the effect on lean body mass was small and non-significant (SMD = 0.13, 95% CI: −0.01, 0.28), suggesting that there is statistical uncertainty in this effect. The difference from the previous meta-analysis results of Lin et al. is that they found that protein supplementation significantly increased lean body mass by 0.32 kg, while our study only found that lean body mass increased by 0.13 kg, and this increase did not reach statistical significance. However, the results obtained by Lin et al. were moderately heterogeneous (*I*^2^ = 38%) (25), and the population included in the study included the elderly and chronic disease groups. In contrast, our study focused more on healthy young and middle-aged populations, which reduced the heterogeneity in the study results due to subject characteristics.

Normally, protein synthesis and breakdown rates in skeletal muscle are balanced, maintaining net protein balance (64). During and after various training types, both processes are stimulated (19). Unlike resistance training, endurance training primarily increases mitochondrial protein concentration without inducing muscle hypertrophy (65). This effect is due to enhanced mitochondrial protein synthesis alongside minimal acute changes in myofibrillar protein synthesis (66). During concurrent training, protein supplementation can augment muscle protein synthesis, helping to offset the catabolic effects of endurance training and promote hypertrophy and strength gains during subsequent resistance training (55, 67). However, this increase is smaller than that from resistance training alone. For example, Walker et al. (49) observed that during the 8-week concurrent training period, the lean body mass of the protein supplement group increased slightly; however, the increase was not as substantial as that observed in the resistance training group. A similar conclusion was reached in the study of Ferguson-Stegall et al. (56) During the 4-week endurance training, the lean body mass of the protein supplement group increased more than that of the carbohydrate group, but the difference was not significant. This indicates that endurance training slightly but not significantly increases lean body mass to a certain extent. One explanation is that the mechanism by which protein supplementation promotes lean body mass increase is likely to be a greater response to myofibrillar protein synthesis after exercise stimulation. Protein supplementation can further promote the rate of myofibrillar muscle protein synthesis (68, 69). In addition, protein supplementation after endurance exercise has also been found to regulate specific mRNA signaling pathways related to muscle synthesis and type I muscle fiber remodeling (70). Another possible explanation is that the protein supplement group may have consumed more total energy (and/or protein) or expended less energy during training compared to the placebo group (49). In addition, the increase in protein intake significantly improved nitrogen balance (49), which helped prevent the loss of muscle mass (10). This process may have led to an increase in muscle mass and total body water (52). It is worth noting that although no significant mediation effect was found in the subgroup analysis of lean body mass, previous studies have shown that the improvement of lean body mass may be affected by factors such as gender and initial lean body mass composition, among which men have a larger increase than women (51), and low initial lean body mass has a larger increase than high lean body mass (50). It may be that due to the current insufficient sample size, no significant mediation effect can be observed. Nevertheless, the small effect size observed in our meta-analysis (SMD = 0.13) and the lack of statistical significance (*p* = 0.07) also confirm the conclusions of previous studies. In conclusion, the effect of protein supplementation on lean body mass during endurance training is small and insignificant.

### 4.2 Physiological adaptation and protein supplementation

#### 4.2.1 Aerobic capacity

Our meta-analysis results showed that protein supplementation had no significant effect on VO_2_max during endurance training (SMD = 0.10, 95% CI: −0.07, 0.27), which is contrary to the previous meta-analysis results of Lin et al. (25), who found that protein supplementation further increased VO_2_max by 0.89 mL/kg/min. However, the number of studies included by Lin et al. (25) was small (only 9). In contrast, we included 19 studies, integrated the latest original experiments, and further explored potential modulators of VO_2_max through subgroup and regression analyses. Previous studies have found that protein supplementation may provide more benefits for untrained subjects, while trained subjects may experience a ceiling effect because their own systematic training has already led to a significant increase in VO_2_max. Consistent with previous studies, the effect size for protein supplementation appeared larger in untrained subjects (SMD = 0.21); however, this difference did not reach statistical significance (*p* = 0.06), indicating uncertainty about the true effect. The major determinants of VO_2_max are the ability of the cardiovascular system to deliver oxygen to working muscles, which is primarily influenced by increases in cardiac output, and the ability of skeletal muscle to extract and utilize oxygen, which is influenced by increases in mitochondrial density and oxidase activity (71). Ferguson-Stegall et al. (56) suggested that the improvement in VO_2_max with protein supplementation was most likely due to cardiovascular adaptation rather than increased oxidative enzyme or mitochondrial biogenesis. This may be because protein supplementation increases plasma albumin levels (72), and hepatic albumin synthesis also increases during endurance training (73, 74). Therefore, protein supplementation may induce greater hepatic albumin synthesis, thereby promoting plasma volume expansion and, in turn, improving VO_2_max. In addition, Knuiman et al. (59) found that protein supplementation increased citrate synthase activity in skeletal muscle, suggesting that protein supplementation may enhance mitochondrial adaptation and thus contribute to the increase in VO_2_max. Nevertheless, there is still insufficient evidence to prove that protein supplementation further improves VO_2_max levels, and several studies (26, 27, 51, 53, 58, 61, 63) have shown no significant difference in VO_2_max between protein-supplemented groups and controls. Under the premise of adequate carbohydrate intake, protein supplementation may enhance molecular markers of training adaptation (e.g., mTOR expression and enzyme activity); however, this does not appear to translate into significant improvements in aerobic capacity. This suggests that although protein supplements can reduce exercise-induced muscle damage, stimulate muscle protein synthesis rate, and improve exercise training adaptability, the improvement of aerobic capacity mainly depends on regular systematic training rather than protein supplements. Owing to methodological differences among studies—such as variation in protein supplementation duration, training volume, and subject characteristics—the effects of protein supplementation on aerobic capacity in endurance athletes remain inconclusive and warrant further investigation. In addition, since the experimental periods included in this article are mostly between 6 and 12 weeks, the low intensity of endurance training and the short supplementation period may also be the reason why no significant changes in VO_2_max were observed in this article.

#### 4.2.2 Anaerobic capacity

Our meta-analysis results showed that protein supplementation had no significant effect on peak power during endurance training (SMD = 0.19, 95% CI: −0.11, 0.49). Considering that there are currently few studies on peak power (only 4) and low statistical power, this result was rated as low level of evidence in GRADE and needs to be treated with caution. Since protein supplementation after exercise can stimulate myofibrillar protein synthesis and help maintain or increase lean body mass, lower limb lean body mass is closely related to peak power output (75). In addition, Reljic et al. (55) showed that the leg muscle strength of the protein supplement group was more significantly improved than that of the control group. Protein intake may increase the power output efficiency per unit muscle mass by increasing the density of contractile proteins in muscle fibers (4). Therefore, it is hypothesized that increases or maintenance of lean body mass might be associated with greater power output, although this relationship requires further investigation. However, there is currently a lack of strong evidence, and more high-quality studies are needed for further verification.

### 4.3 Performance and protein supplementation

Our meta-analysis results showed that protein supplementation had no significant effect on TT and sprint speed during endurance training, but significantly increased TTE (SMD = 0.45, 95% CI: 0.15–0.76). Unlike the previous meta-analysis by Lin et al. (25), which found that protein supplementation could further improve TT (MD = −29.1 s), our study did not observe a statistically significant effect on TT, but found significant benefits in TTE. This difference may be related to factors such as the type of included studies, training protocols, subject characteristics, or assessment methods. In terms of TT and sprint speed, it is not surprising that there was no significant effect after protein supplementation, considering that most of the included studies were well-trained or trained endurance athletes, who may have had high baseline levels. Secondly, the four studies (26, 49, 59, 61) showed that the differences in performance observed in the current studies may be related to the length of the experiment and the volume and frequency of endurance exercise training. The poor effect of protein supplementation may be related to the low volume and frequency of endurance exercise training or the short duration of the study. Previous studies (76) have shown a trend toward improved 5 km timed run performance with a high-protein diet compared with a low-protein diet (*p* = 0.06), but these short-term studies did not translate into long-term benefits. Therefore, the beneficial effects of protein supplementation on exercise performance during long-term endurance training remain to be determined.

One potential mechanism by which protein supplementation significantly improves exhaustion time may be related to its enhancement of aerobic metabolism. Studies have shown that protein supplements can further stimulate fat oxidation during exercise, thereby delaying glycogen depletion and improving endurance performance (77). In addition, the improvement of endurance performance not only depends on aerobic and anaerobic metabolic capacity, but is also closely related to neuromuscular function and running economy (78, 79). Studies have found that post-exercise protein intake can induce stronger activation of anabolic signaling pathways (such as mTOR) in human skeletal muscle within 4 h after acute resistance training, a process that may bring long-term adaptation to muscle-tendon structure (80). Therefore, it can be speculated that protein supplementation may improve running economy by promoting positive remodeling of muscle-tendon structure and mechanical properties (81), allowing athletes to achieve higher maximum running speeds at the same relative oxygen consumption (82). However, this hypothesis still needs to be verified by more studies focusing on structural adaptation and running economy mechanisms. In addition, it should be noted that the TTE test differs substantially from real-world competitive scenarios, and thus, the practical significance of improvements in TTE should be interpreted with caution.

### 4.4 Practical implications

This study used meta-analysis techniques to systematically review the effects of endurance training combined with protein supplementation on body composition, physiological adaptations, and exercise performance to update previous research conclusions, provide practitioners with the latest integrated results, and focus on the interaction between endurance training and protein supplementation. Our study showed that protein supplementation during endurance training significantly improved the TTE and slightly increased the lean body mass. It is worth noting that considering that previous studies have shown that the current literature still lacks studies exploring the role of protein supplementation in long-term endurance training (83), we limited inclusion to studies with supplementation periods longer than 4 weeks. However, this may affect the generalizability of our findings. However, in real life, researchers and practitioners are more concerned about the long-term adaptation of protein supplementation to endurance athletes (15). In terms of daily protein intake, the average range of the studies we included was 1.2 g/kg/day to 2.3 g/kg/day, which meets the dose recommended by the current nutrition association. During training, higher protein intake can meet the needs of cellular protein synthesis and metabolism (13, 84), and the daily protein intake of endurance athletes usually reaches the habitual intake level (24). For example, endurance-trained athletes need about 1.8 g/kg/day to support endurance training adaptation (85). Nonetheless, practitioners need to take into account metabolic differences between different endurance sports as well as individual differences (86), all of which will affect protein requirements.

### 4.5 Limitation

Before interpreting the results of this paper, some limitations need to be clarified. First, only peer-reviewed and published literature was searched in the literature search, but there was no language restriction. This ensured the quality of the included articles to a certain extent, but there may still be risks of selection and publication bias. Second, most studies did not report the habitual protein intake of the subjects, which resulted in our inability to directly evaluate whether individuals undergoing endurance training benefited from additional protein. Third, we used the SD of mean change in the selection of SD. Since the correlation coefficients before and after the intervention were rarely reported in the included studies, we conservatively assumed that the correlation coefficient *r* = 0.50, which is also in line with the recommendations of the Cochrane guidelines. Although this assumption is a conservative estimate, its deviation from the true correlation coefficient may have a certain impact on the effect size estimate. Fourth, the inconsistent reporting of protein supplementation dosage in the included studies limits our comprehensive evaluation of the moderating effect of supplementation dosage.

### 4.6 Prospect

First, it is recommended that future studies clarify the habitual protein intake of subjects and establish a control group that consumes a caloric placebo, which will help evaluate whether the adaptations to endurance training are attributed to protein supplementation. Second, although our study included sedentary healthy people to well-trained athletes, it is necessary to explore the feasibility and physiological adaptations of protein supplementation in chronic disease populations (such as diabetic patients) and sarcopenia populations. Third, the small sample size, lack of large-scale long-term studies, and insufficient representation of female participants are limitations of the current study. Future studies need to further expand the sample size and investigate the physiological adaptations brought about by protein supplementation during endurance training to clarify the level of contribution of protein supplementation to performance in specific populations. Fourth, there is currently a lack of experimental studies to explore whether the addition of protein can further promote muscle glycogen recovery when consuming optimal carbohydrate intake, which is of great significance for formulating recovery nutrition strategies for endurance athletes.

## 5 Conclusion

Protein supplementation during endurance training results in a small, non-significant increase in lean body mass and significantly improves TTE during endurance exercise. Protein supplementation does not cause significant changes in body weight and body fat, nor does it affect aerobic and anaerobic capacity, but untrained adults may further improve VO_2_max with protein supplementation compared to trained adults.

## Data Availability

The original contributions presented in the study are included in the article/Supplementary material, further inquiries can be directed to the corresponding author.

## Footnotes

^1^ https://apps.automeris.io/wpd4/
