# Supplementary material for: Effects of protein supplementation on body composition, physiological adaptations, and performance during endurance training: a systematic review and meta-analysis
Source: Front Nutr. 2025 Aug 7;12:1663860. doi: 10.3389/fnut.2025.1663860 (PMC12369418; doi:10.3389/fnut.2025.1663860)
Supplement: Supplementary file 1 [file Data_Sheet_1.zip › Appendix S1 Complete search strategy for all databases.docx]

**Appendix S1** Complete search strategy for all databases

| Database | Query | Results |
| --- | --- | --- |
| PubMed | "Physical Endurance"[MeSH Terms] OR "Exercise Training"[All Fields] OR "endurance training"[All Fields] OR "aerobic training"[All Fields] OR "aerobic exercise"[All Fields] OR "cardiorespiratory training"[All Fields] OR "continuous training"[All Fields] OR "stamina training"[All Fields] OR "endurance exercise"[All Fields] #1  "Dietary Proteins"[MeSH Terms] OR "Whey Proteins"[MeSH Terms] OR "Caseins"[MeSH Terms] OR "Amino Acids"[MeSH Terms] OR "branched-chain amino acids"[All Fields] OR "protein supplementation"[All Fields] OR "protein supplement"[All Fields] OR "whey protein"[All Fields] OR "casein"[All Fields] OR "protein intake"[All Fields] OR "amino acid supplementation"[All Fields] OR "branched-chain amino acids"[All Fields] OR "protein powder"[All Fields] OR "protein shakes"[All Fields] #2  "Athletic Performance"[MeSH Terms] OR "Physical Performance"[All Fields] OR "Exercise Test"[MeSH Terms] OR "Endurance"[All Fields] OR "Physical Fitness"[MeSH Terms] OR "Sports"[MeSH Terms] OR "exercise performance"[All Fields] OR "Athletic Performance"[All Fields] OR "sports performance"[All Fields] OR "endurance performance"[All Fields] OR "VO2max"[All Fields] OR "time trial performance"[All Fields] OR "physical capacity"[All Fields] OR "fatigue resistance"[All Fields] #3  "Body Composition"[MeSH Terms] OR "Lean Body Mass"[All Fields] OR "muscle, skeletal"[MeSH Terms] OR "fat free mass"[All Fields] OR "Body Fat Distribution"[MeSH Terms] OR "Body Weight"[MeSH Terms] OR "Body Composition"[All Fields] OR "Lean Body Mass"[All Fields] OR "fat free mass"[All Fields] OR "muscle mass"[All Fields] OR "skeletal muscle mass"[All Fields] OR "body fat"[All Fields] OR "body fat percentage"[All Fields] OR "adiposity"[All Fields] OR "fat mass"[All Fields] OR "BMI"[All Fields] #4  "randomized controlled trial"[Publication Type] OR "Randomized"[Title/Abstract] OR "randomised"[Title/Abstract] OR "RCT"[Title/Abstract] #5  #1+#2+#3+#4+#5 | 297 |
| Web of Science | **TS=("endurance training" OR "aerobic training" OR "aerobic exercise" OR "cardiorespiratory training" OR "continuous training" OR "stamina training" OR "endurance exercise" OR "exercise training" OR "physical endurance") #1**  **TS=("dietary protein" OR "whey protein" OR "casein" OR "amino acids" OR "branched-chain amino acids" OR "protein supplementation" OR "protein supplement" OR "protein intake" OR "amino acid supplementation" OR "protein powder" OR "protein shakes") #2**  **TS=("athletic performance" OR "physical performance" OR "exercise test" OR "endurance" OR "physical fitness" OR "sports" OR "exercise performance" OR "sports performance" OR "endurance performance" OR "VO2max" OR "time trial performance" OR "physical capacity" OR "fatigue resistance") #3**  **TS=("body composition" OR "lean body mass" OR "fat free mass" OR "muscle mass" OR "skeletal muscle mass" OR "body fat" OR "body fat percentage" OR "adiposity" OR "fat mass" OR "body weight" OR "body fat distribution" OR "BMI" OR "skeletal muscle") #4**  **TS=(randomized OR randomised OR RCT) #5**  #1+#2+#3+#4+#5 | 118 |
| SPORTDiscus | "Body Composition" OR "Lean Body Mass" OR "Muscle, Skeletal" OR "Fat-Free Mass" OR "Body Fat Distribution" OR "Body Weight" OR "body composition" OR "lean body mass" OR "fat-free mass" OR "muscle mass" OR "skeletal muscle mass" OR "body fat" OR "body fat percentage" OR "adiposity" OR "fat mass" OR "BMI" #1  "Athletic Performance" OR "Physical Performance" OR "Exercise Test" OR "Endurance" OR "Physical Fitness" OR "Sports" OR "exercise performance" OR "athletic performance" OR "sports performance" OR "endurance performance" OR "VO2max" OR "time trial performance" OR "physical capacity" OR "fatigue resistance" #2  "Physical Endurance" OR "Exercise Training" OR "endurance training" OR "aerobic training" OR "aerobic exercise" OR "cardiorespiratory training" OR "continuous training" OR "stamina training" OR "endurance exercise" #3  "Dietary Proteins" OR "Whey Proteins" OR "Caseins" OR "Amino Acids" OR "Branched-Chain Amino Acids" OR "protein supplementation" OR "protein supplement" OR "whey protein" OR "casein" OR "protein intake" OR "amino acid supplementation" OR "branched-chain amino acids" OR "protein powder" OR "protein shakes" #4  "Randomized Controlled Trial" OR "randomized" OR "randomised" OR "RCT" #5  #1+#2+#3+#4+#5 | 35 |
| Overall | n/a | 450 |
