# Supplementary material for: Effects of protein supplementation on body composition, physiological adaptations, and performance during endurance training: a systematic review and meta-analysis
Source: Front Nutr. 2025 Aug 7;12:1663860. doi: 10.3389/fnut.2025.1663860 (PMC12369418; doi:10.3389/fnut.2025.1663860)
Supplement: Supplementary file 1 [file Data_Sheet_1.zip › Appendix S3.docx]

**Appendix S3.** Methodological quality assessment (PEDro)

| Author, year | D1 | D2 | D3 | D4 | D5 | D6 | D7 | D8 | D9 | D10 | D11 | Total |
| --- | --- | --- | --- | --- | --- | --- | --- | --- | --- | --- | --- | --- |
| Antonio et al - 2000 | Y | 1 | 0 | 1 | 1 | 0 | 1 | 1 | 0 | 1 | 1 | 7 |
| Flakoll et al - 2004 | Y | 1 | 0 | 1 | 1 | 0 | 1 | 1 | 0 | 1 | 1 | 7 |
| Lockwood et al，2008 | Y | 1 | 0 | 1 | 0 | 0 | 0 | 1 | 0 | 1 | 1 | 5 |
| Walker et al，2010 | Y | 1 | 0 | 1 | 1 | 0 | 1 | 1 | 0 | 1 | 1 | 7 |
| Ferguson-Stegall et al，2011 | Y | 1 | 0 | 1 | 1 | 0 | 1 | 1 | 0 | 1 | 1 | 7 |
| Cramer et al，2012 | Y | 1 | 0 | 1 | 1 | 0 | 1 | 1 | 0 | 1 | 1 | 7 |
| Berg et al，2012 | Y | 1 | 1 | 1 | 0 | 0 | 0 | 1 | 0 | 1 | 1 | 6 |
| Gryson et al，2014 | Y | 1 | 0 | 1 | 1 | 0 | 1 | 1 | 0 | 1 | 1 | 7 |
| McAdam et al，2018 | Y | 1 | 0 | 1 | 1 | 0 | 1 | 1 | 1 | 1 | 1 | 8 |
| Ormsbee et al - 2018 | Y | 1 | 0 | 1 | 0 | 0 | 0 | 1 | 0 | 1 | 1 | 5 |
| Jonvik et al - 2019 | Y | 1 | 1 | 1 | 1 | 0 | 1 | 1 | 0 | 1 | 1 | 8 |
| Knuiman et al-2019 | Y | 1 | 1 | 1 | 1 | 0 | 0 | 1 | 1 | 1 | 1 | 8 |
| Naclerio et al - 2019 | Y | 1 | 0 | 1 | 1 | 0 | 1 | 1 | 0 | 1 | 1 | 7 |
| Forbes et al - 2020 | Y | 1 | 0 | 1 | 0 | 0 | 0 | 1 | 0 | 1 | 1 | 5 |
| Hansen et al - 2020 | Y | 1 | 0 | 1 | 1 | 0 | 1 | 1 | 0 | 1 | 1 | 7 |
| Alghannam et al - 2020 | Y | 1 | 0 | 1 | 1 | 0 | 1 | 1 | 0 | 1 | 1 | 7 |
| Jendricke et al - 2020 | Y | 1 | 0 | 1 | 1 | 0 | 1 | 1 | 0 | 1 | 1 | 7 |
| Röhling et al - 2021 | Y | 1 | 0 | 1 | 0 | 0 | 0 | 1 | 0 | 1 | 1 | 5 |
| Hsu et al - 2021 | Y | 1 | 0 | 1 | 0 | 0 | 0 | 1 | 1 | 1 | 1 | 6 |
| Valenzuela et al - 2021 | Y | 1 | 1 | 1 | 1 | 1 | 0 | 1 | 1 | 1 | 1 | 9 |
| Li et al - 2021 | Y | 1 | 0 | 1 | 0 | 0 | 0 | 1 | 0 | 1 | 1 | 5 |
| Jerger et al - 2023 | Y | 1 | 0 | 1 | 1 | 0 | 1 | 1 | 0 | 1 | 1 | 7 |
| Reljic et al - 2024 | Y | 1 | 0 | 1 | 1 | 0 | 1 | 1 | 0 | 1 | 1 | 7 |

Note***:*** studies scoring ≥6 are considered **high quality**, those scoring 4-5 are considered **moderate quality**, and those scoring <4 are considered **low quality**.

1. eligibility criteria were specified (not included in the total score)

2. subjects were randomly allocated to groups (in a crossover study, subjects were randomly allocated an order in which treatments were received)

3. allocation was concealed

4. the groups were similar at baseline regarding the most important prognostic indicators

5. there was blinding of all subjects

6. there was blinding of all therapists who administered the therapy

7. there was blinding of all assessors who measured at least one key outcome

8. measures of at least one key outcome were obtained from more than 85% of the subjects initially allocated to groups

9. all subjects for whom outcome measures were available received the treatment or control condition as allocated or, where this was not the case, data for at least one

key outcome was analysed by “intention to treat”

10. the results of between-group statistical comparisons are reported for at least one key outcome

11. the study provides both point measures and measures of variability for at least one key outcome
