# Supplementary figures and images for: Effects of protein supplementation on body composition, physiological adaptations, and performance during endurance training: a systematic review and meta-analysis
Source: Front Nutr. 2025 Aug 7;12:1663860. doi: 10.3389/fnut.2025.1663860 (PMC12369418; doi:10.3389/fnut.2025.1663860)

**Appendix S4.** Funnel plot of publication bias


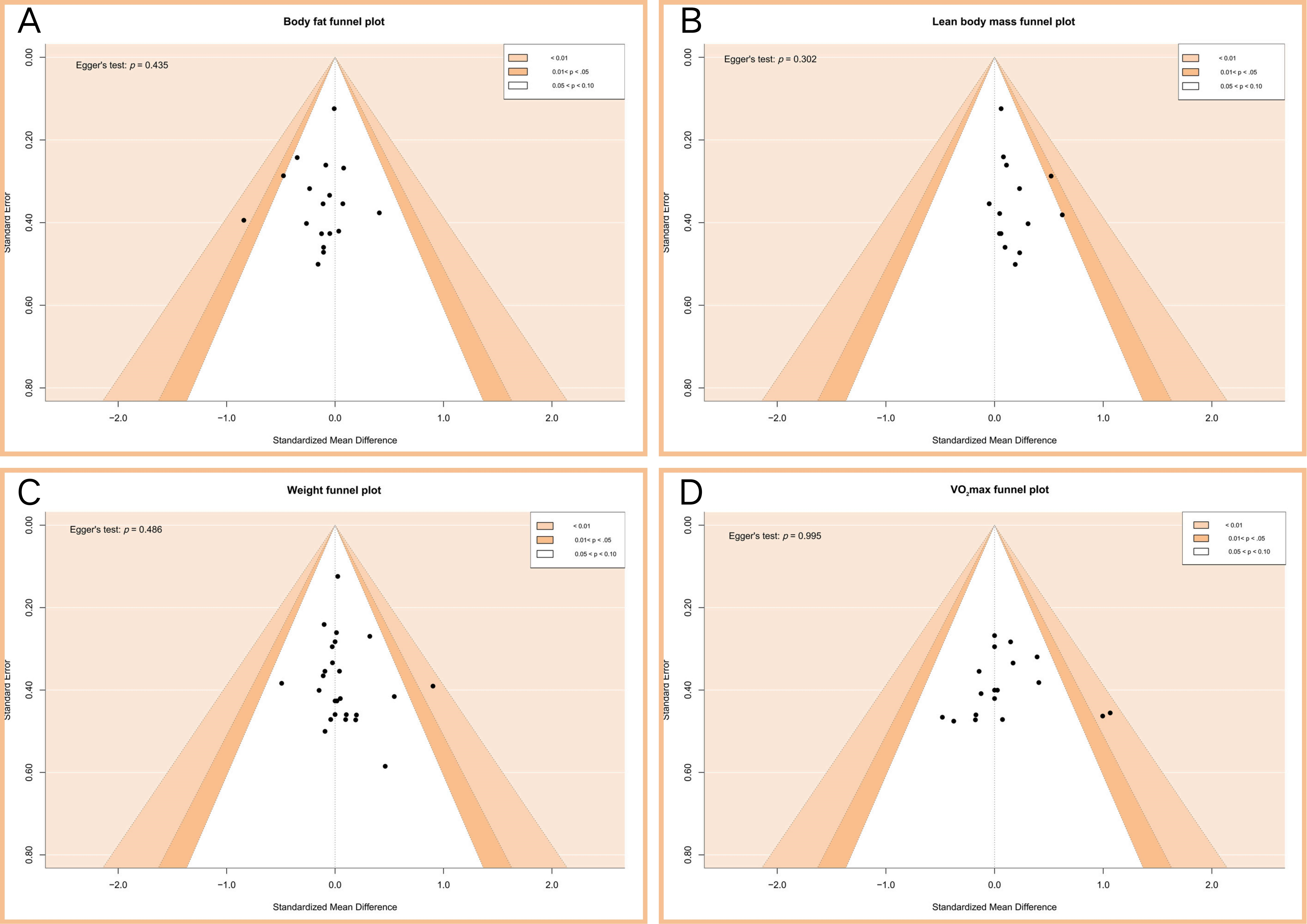

Supplement: Supplementary file 1 [file Data_Sheet_1.zip › Appendix S4.docx]

**Appendix S2.** Risk of bias of the studies

**
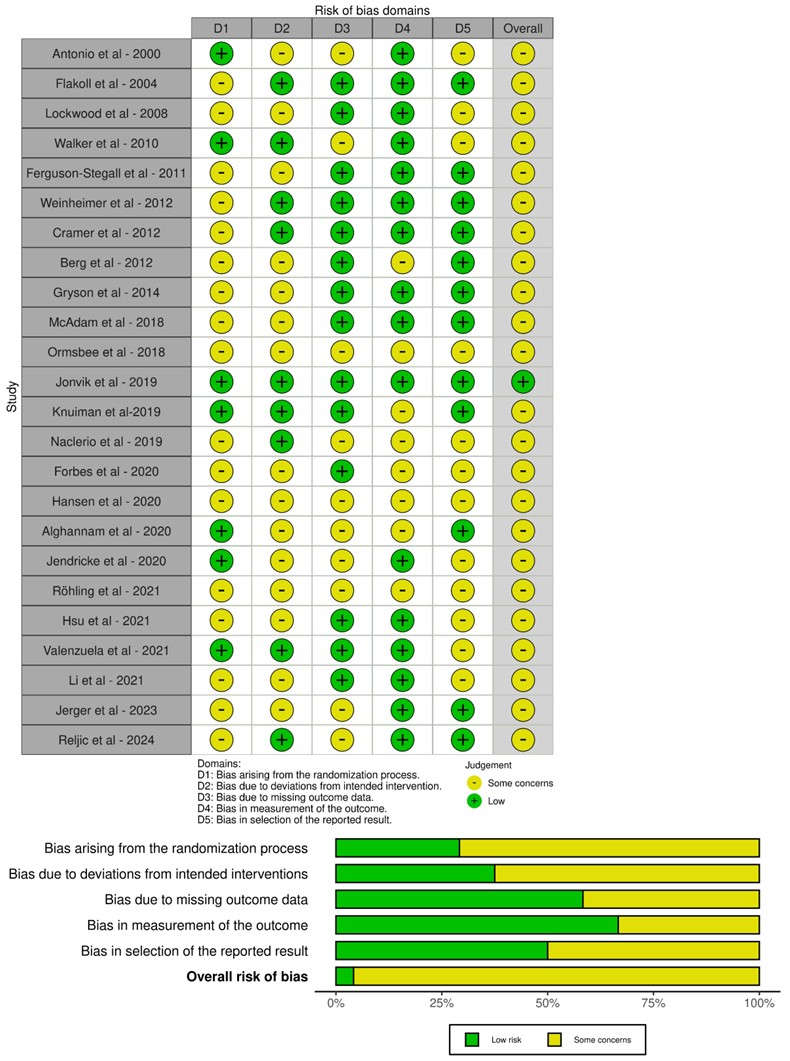
**

Supplement: Supplementary file 1 [file Data_Sheet_1.zip › Appendix S2.docx]
